# Four-Dimensional Computed Tomography of Respiratory Function Changes Post-Radiotherapy for Lung Cancer

**DOI:** 10.7759/cureus.103971

**Published:** 2026-02-20

**Authors:** Shiho Wada, Tomohiro Itonaga, Ryuji Mikami, Tatsuhiko Zama, Yukinori Okada, Kazuhiro Saito

**Affiliations:** 1 Department of Radiology, Tokyo Medical University Hospital, Tokyo, JPN

**Keywords:** 4dct, lung cancer, radiotherapy, respiratory, rili, ventilation

## Abstract

Radiotherapy (RT) plays an important role in the curative treatment of lung cancer; however, longitudinal changes in regional pulmonary function after treatment remain incompletely understood. In this case report, we present a single-patient, hypothesis-generating analysis evaluating the association between lung dose and regional functional changes using deformable image registration (DIR)-based four-dimensional computed tomography (4DCT) ventilation imaging (Jacobian-derived) obtained at three time points: pre-RT, six months post-RT, and one year post-RT. An 83-year-old male with clinical stage IIIA right upper lobe lung cancer underwent definitive chemoradiotherapy. Consolidation durvalumab was initiated post-treatment, and the patient developed pneumonitis consistent with radiation-induced lung injury (Common Terminology Criteria for Adverse Events (CTCAE) grade 2), which was managed with oral prednisolone. SpO₂ was 94% on room air, and oxygen therapy was not required. Dynamic analysis of 4DCT images showed that the volume of the highly irradiated right upper lobe decreased over time, while the rate of contraction increased. The volume of the non-irradiated left lower lobe increased by 11% at one year post-RT, whereas the rate of contraction decreased slightly. 4DCT-derived ventilation suggested a dose-related decline in ventilation that persisted at one year, including within low-dose regions. This single-case observation supports the potential value of integrating functional information with anatomic CT findings during post-RT follow-up and in efforts to preserve functional lung, while acknowledging the limitations related to a single patient, DIR-dependent ventilation estimates, and the absence of post-treatment pulmonary function testing.

## Introduction

Radiotherapy (RT) and surgery play important roles in the curative treatment of non-small cell lung cancer (NSCLC). However, there are many differences in the approach to assess changes in respiratory function pre- and post-treatment. Guidelines for thoracic surgery recommend that the decision to operate on patients with lung cancer should be based on preoperative pulmonary function tests (PFTs) and the number of lobes to be resected [1]. In contrast, guidelines for RT place less emphasis on preoperative PFTs and state the importance of reducing the parenchymal volume (VX) of the lung that has received X Gy [2]. This difference may be because surgery reduces respiratory function consistent with the loss of anatomical structures, whereas RT leads to changes in respiratory function over time due to fibrosis and inflammation in the lung, consistent with the irradiated area.

Studies have been conducted on changes in respiratory function post-RT, but most have used ventilation and perfusion single-photon emission computed tomography (V/P SPECT) [3]. V/P SPECT has problematic invasiveness and temporal and spatial resolution, and in recent years, there has been a focus on imaging of lung ventilation using four-dimensional computed tomography (4DCT). This imaging technique adds a time axis to 3DCT and continuously acquires CT images while recording the respiratory waveform; a respiratory cycle is divided into 10 phases, with each image corresponding to a specific respiratory phase. By reconstructing the data, it is possible to visualize, in detail, the dynamics of the organs during respiration. In this single-case, exploratory report, we longitudinally evaluated dose-overlaid 4DCT ventilation (Jacobian-derived) at three time points (pre-RT, six months post-RT, and one-year post-RT) after definitive chemoradiotherapy for stage III NSCLC, with lobar volume/contractility and contralateral changes presented as supportive anatomic context. Because 4DCT ventilation estimates are deformable image registration (DIR)-dependent, the results may be influenced by methodological choices and should be interpreted as hypothesis-generating.

An earlier version of this article was posted to the Authorea preprint server on March 28, 2025 (doi: 10.22541/au.174313815.53650464/v1).

## Case presentation

**Figure 1:**
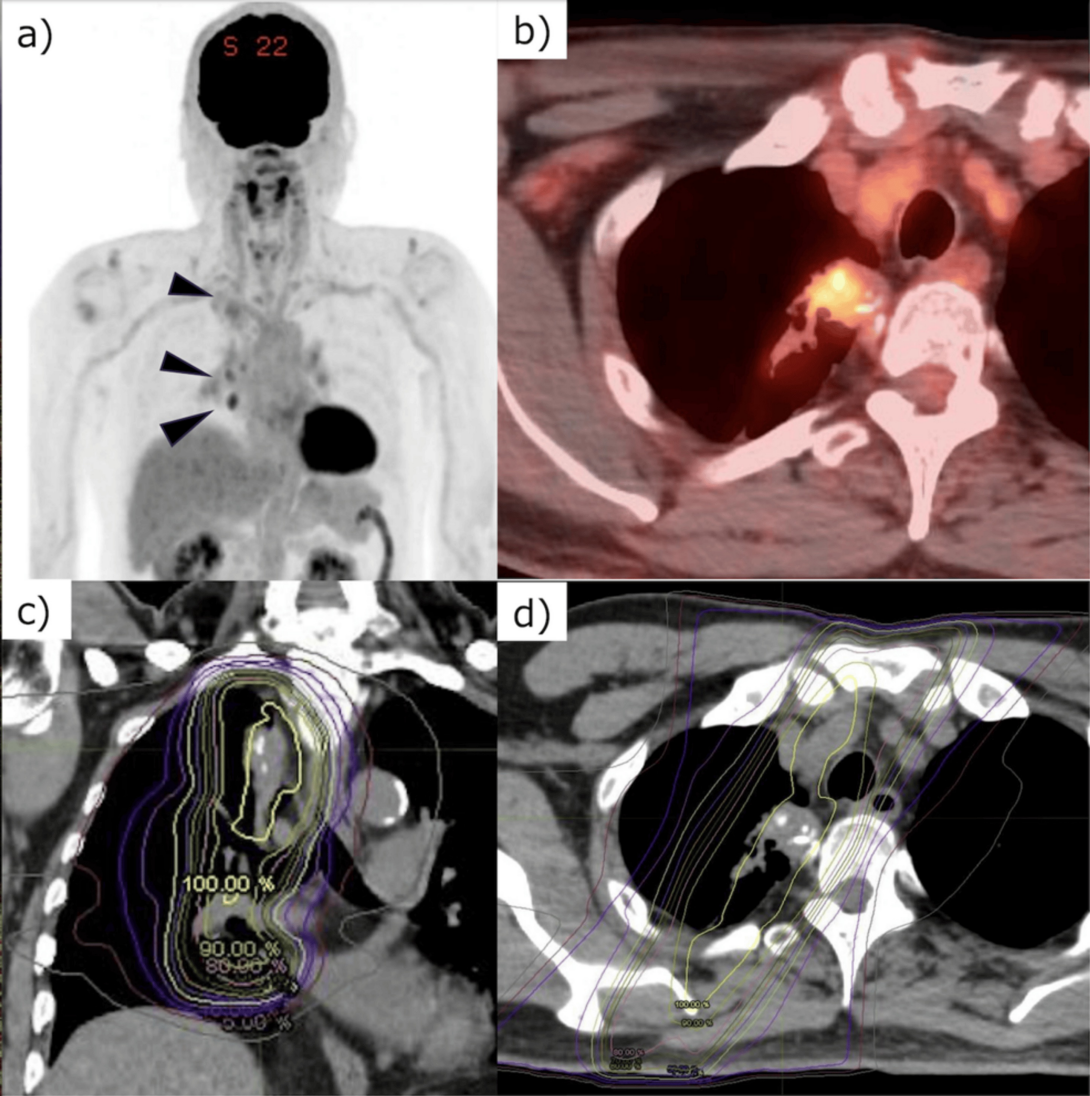
A treatment plan for clinical stage T2aN2M0 right upper lobe lung cancer using three-dimensional conformal radiotherapy 18-Fluorine fludeoxyglucose positron emission tomography/computed tomography (PET/CT) was performed for lung cancer staging in an 83-year-old male. (a) Maximum intensity projection image showing accumulation in the right upper lobe and ipsilateral mediastinal lymph nodes (black arrow heads). (b) Axial image of the primary tumor on PET/CT, and the biopsy confirmed an adenocarcinoma. A treatment plan was developed for the clinical stage T2aN2M0 right upper lobe lung cancer using three-dimensional conformal radiotherapy. The total dose was 66 Gy in 33 fractions, and the treatment plan was modified at 40 Gy to reduce the dose to the spine and lungs. Panels (c) and (d) show coronal and axial sections of the combined first and second plan dose distribution maps.

An 83-year-old male with stage IIIA (clinical stage cT2aN2M0) right upper lobe NSCLC underwent chemoradiotherapy as the definitive treatment. He was an ex-smoker (20 cigarettes/day for 13 years, ages 20-33) and had no known chronic obstructive pulmonary disease (COPD) or interstitial lung disease; baseline dyspnea was not present. Baseline PFTs were obtained pre-treatment (slow vital capacity (SVC) 3.08 L, inspiratory reserve volume (IRV) 1.94 L, inspiratory capacity (IC) 2.45 L, tidal volume (TV) 0.51 L, expiratory reserve volume (ERV) 0.63 L, forced expiratory volume in 1 second (FEV1.0) 3.08 L, and FEV1.0% 62.66%). The patient received RT using three-dimensional conformal RT with 66 Gy in 33 fractions administered once daily and concurrent chemotherapy with weekly carboplatin and paclitaxel (Figure 1).​​​​​​​

For treatment planning, 4DCT was used to map the respiratory migration of the tumor. 4DCT was performed with a 16-row multidetector CT (Aquilion LB, Canon Medical Systems Corp., Otawara, Japan) using a respiratory synchronization system (Respiratory Gating for Scanners; Varian Medical Systems, Palo Alto, CA). Dosimetric parameters for treatment planning were 25.1% for V20, representing the percentage of total lung volume irradiated with ≥20 Gy, and 46.5% for V5. No serious adverse events occurred during chemoradiotherapy, and an immune checkpoint inhibitor (ICI) was subsequently administered. Durvalumab was initiated approximately four weeks after completion of chemoradiotherapy (1500 mg/body every four weeks). After cycle 1, Common Terminology Criteria for Adverse Events (CTCAE) grade 2 pneumonitis consistent with radiation-induced lung injury was observed; oral prednisolone was started, and durvalumab was held.Durvalumab was later restarted from cycle 2, while the patient was on low-dose prednisolone and was subsequently continued to a total of 12 cycles. To ensure consistency across time points, follow-up 4DCT scans at six months and one year post-RT were acquired using the same protocol and positioning as the planning scan (supine, both arms raised above the head, without an immobilization device). Each respiratory cycle was reconstructed into 10 phases (0%-90%), and analyses were performed using the end-inhalation and end-exhalation phases. Scans with markedly irregular breathing were excluded based on respiratory waveform review. One-year post-RT and the eighth course of ICI, there was no evidence of recurrence or metastases. The clinical course and management are summarized in Table 1.

**Table 1:** Clinical timeline of chemoradiotherapy, durvalumab consolidation, pneumonitis management, and serial 4DCT acquisitions cCRT: Concurrent chemoradiotherapy; RT: Radiotherapy; ICI: Immune checkpoint inhibitor; PSL: Prednisolone; 4DCT: Four-dimensional computed tomography; CT: Computed tomography; SpO₂: Peripheral oxygen saturation; PFT: Pulmonary function test; Vjac: Jacobian-based ventilation; CTCAE: Common Terminology Criteria for Adverse Events; RILI: Radiation-induced lung injury.

Timepoint	Clinical event	Management and remarks
Week 1	Baseline assessment	Baseline 4DCT performed (pre-treatment), baseline PFT performed (pre-treatment only)
Week 0	cCRT initiation	Concurrent chemoradiotherapy started.
Week 7	cCRT completion	Radiotherapy completed (66 Gy in 33 fractions).
Week 11	Consolidation therapy	Durvalumab initiated (1500 mg/body, every 4 weeks). Cycle 1 administered.
Week 15	Pneumonitis/RILI onset	Mild dyspnea; CTCAE grade 2. SpO₂ 94% on room air, no oxygen requirement. CT showed changes consistent with RILI within the irradiated field. Durvalumab was held after cycle 1. Prednisolone (PSL) 30 mg/day initiated.
Week 16	Steroid taper attempt	PSL reduced to 15 mg/day; subsequent radiographic worsening noted.
Week 19	Steroid re-escalation	PSL increased back to 30 mg/day due to worsening imaging findings.
Week 21	Clinical improvement	Dyspnea resolved; PSL reduced to 25 mg/day.
Weeks 23–31	Steroid tapering phase	PSL tapered stepwise (20 → 15 → 10 → 5 mg/day).
Week 26	Follow-up imaging	6-month follow-up 4DCT performed.
Week 33	ICI re-challenge	Durvalumab restarted (cycle 2) while on PSL 5 mg/day.
Week 36	Further taper	PSL reduced to 2.5 mg/day.
Week 44	Steroid cessation	PSL discontinued. Managed entirely as an outpatient; no antibiotics and no hospitalization.
Week 57	Follow-up imaging	1-year follow-up 4DCT performed.
Week 75	Treatment completion	Durvalumab completed (cycle 12).

Results

**Figure 2:**
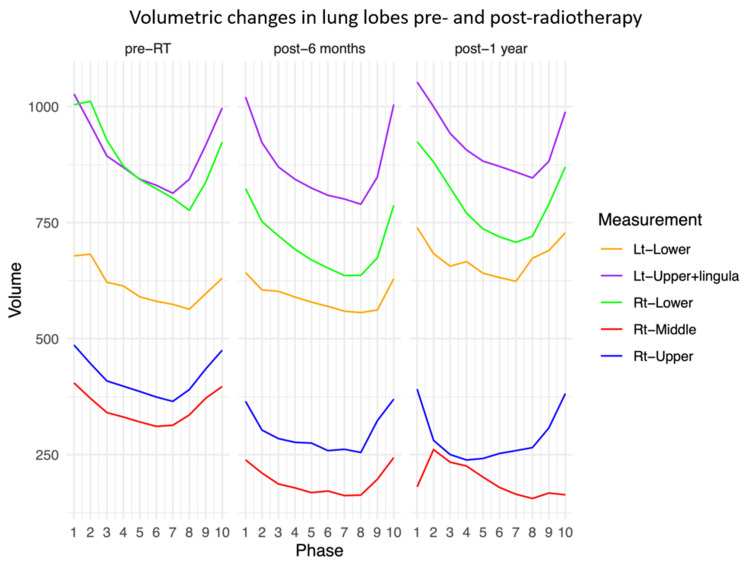
The changes in volume during the respiratory phase for each lung lobe pre- and post-radiotherapy The changes in volume during the respiratory phase for each lung lobe pre- and post-radiotherapy are summarized. The volumes of the right upper and middle lobes, which were within the radiation field, decreased over time, while there was an increase in the left lower lobe one year post-radiotherapy, which was not irradiated.

Dynamic analysis was performed using 4DCT images obtained at three time points; pre-RT, six months post-RT, and one year post-RT. Lung lobe volumes per respiratory phase were measured using Synapse Vincent system lung analysis software (Fujifilm Medical Co., Ltd., Tokyo, Japan). The median total lung volume was 3,121.6 cc pre-RT, 2,593.8 cc six months post-RT and 2,823.3 cc one year post-RT. The changes over time for each lung lobe are summarized as line graphs in Figure 2.

In the middle lobe of the right lung, respiratory changes six months post-RT were normal, whereas one year post-RT, paradoxical respiration was present, driven by fibrosis in the upper lobe. To quantify the extent to which the lung lobes contract during respiration, the contracted volume (CV) was defined as the maximum volume minus the minimum volume, and the contracted index (CI) was defined as the CV divided by the median volume of the lung lobes (Table 2).

**Table 2:** Contraction volume and index pre- and post-treatment for each lung lobe The top row shows CV, and the bottom row shows CI. CV: Contracted volume; CI: Contracted index; RT: Radiotherapy.

Time point	Total lung	Right lung	Right upper	Right middle	Right lower	Left lung	Left upper + Lingular	Left lower
Pre-RT	732.5cc	413.9cc	121.5cc	93.6cc	235cc	318.5cc	214cc	118.6cc
	23%	26%	30%	28%	27%	21%	24%	20%
Six months post-RT	690.3cc	373cc	115cc	81.9cc	187.4cc	317.3cc	231cc	86.2cc
	27%	32%	41%	45%	27%	22%	27%	15%
One year post-RT	675.2cc	365.1cc	152.8cc	105.4cc	216.6cc	310.1cc	206.7cc	116.1cc
	24%	29%	58%	58%	28%	20%	23%	17%

The volume of the right upper lobe, where the primary lesion was located, decreased over time, post-RT, whereas the CI showed an increasing trend. The CV, six months post-RT, indicated a worsening of the effects of radiation pneumonitis (RP), whereas at one year post-RT, there was an increase in CV compared with the CV pre-RT. This may reflect compensatory changes in the non-fibrotic areas of the right upper lobe. The volume of the left lung outside the irradiated area increased compared to the volume pre-RT.

**Figure 3:**
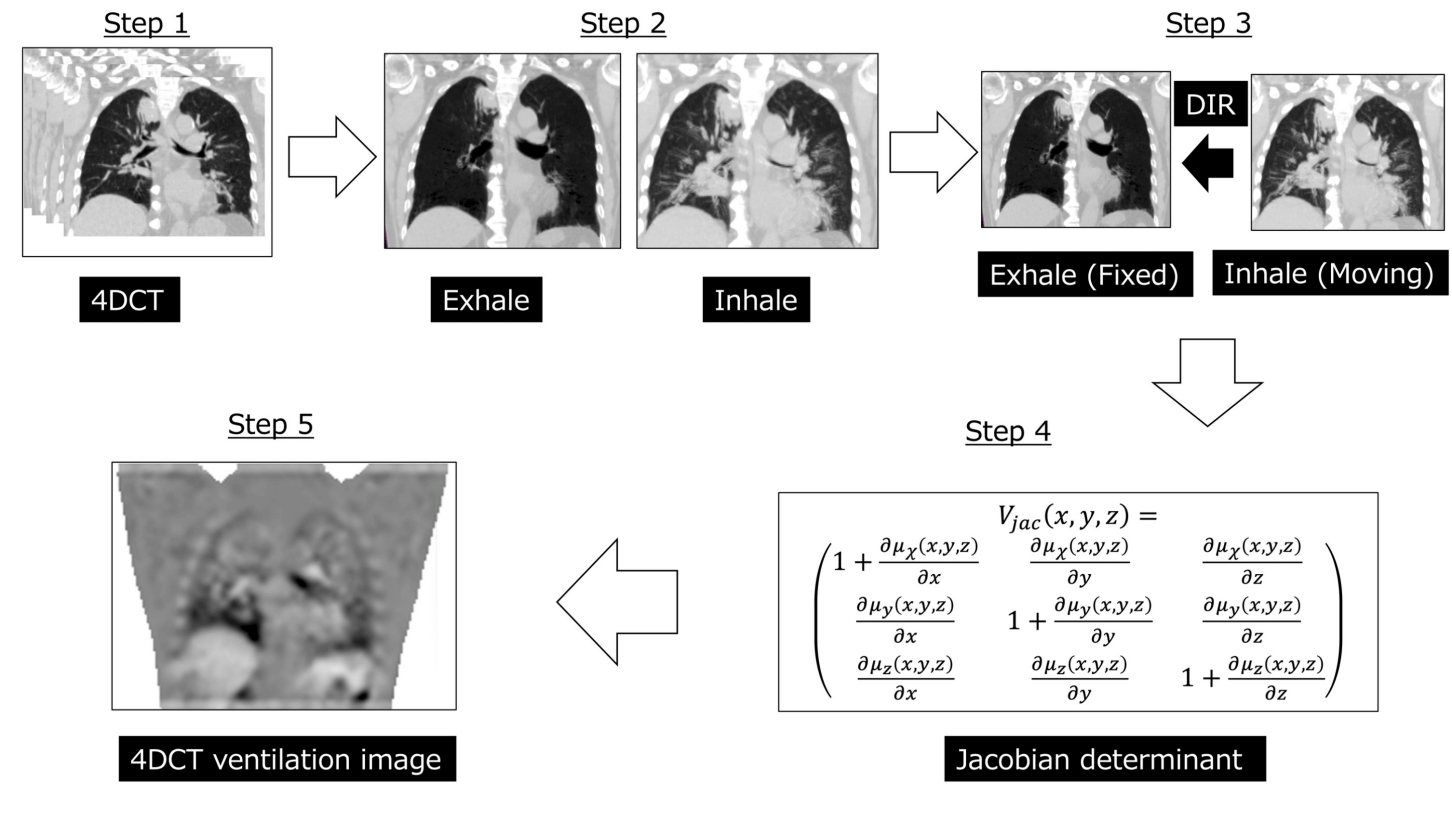
Four-dimensional computed tomography (4DCT) for pulmonary ventilation imaging Four-dimensional computed tomography (4DCT) for pulmonary ventilation imaging is divided into five steps. Step 1: 4DCT is performed; the respiratory cycle is divided into 10 phases, and the image corresponding to each respiratory phase is reconstructed. Step 2: This step is performed using the MIM Maestro treatment planning support device (MIM Software Inc., Cleveland, Ohio). Maximum inspiration and expiration images are generated from the 4DCT images. Step 3: The maximum expiration image is designated as the primary; the maximum inspiration image is designated as the secondary; and DIR is performed to calculate the displacement vector field (DVF) for each voxel in the lung. Step 4: The rate of change in the volume of each voxel is calculated from the DVF using the Jacobian matrix. This method is based on the principle that voxels with a higher change in volume correspond to higher lung function. Step 5: The 4DCT ventilation image is generated, as shown, with black areas representing areas that are dilated (>1) and white areas representing areas that are contracted (<1). The primary lesion in the right upper lobe appears brighter than the contralateral side, indicating reduced ventilation.

Lung ventilation images used to quantify lung ventilation volumes were created from 4DCT images according to a previous study using MIM Maestro (MIM Software Inc., Cleveland, Ohio) [4]. Figure 3 shows the procedure for generating lung ventilation images from 4DCT images.

**Figure 4:**
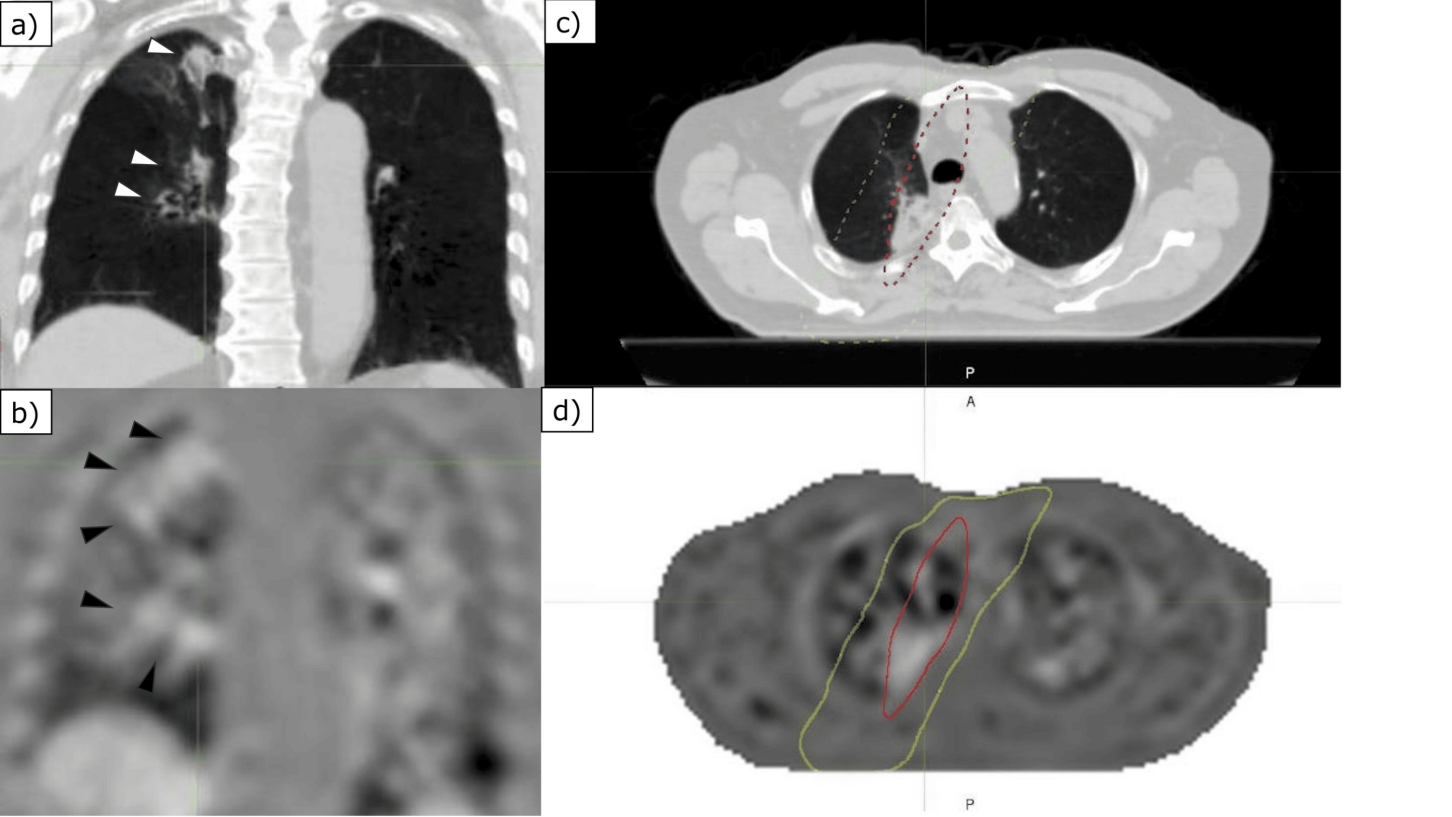
Comparison of four-dimensional computed tomography (4DCT) images and pulmonary ventilation images acquired one year post-treatment (a) Coronal images show the appearance of consolidation (white arrowhead) on the mediastinal side of the right lung in line with the irradiated field. (b) The corresponding lung ventilation image shows a wider range of hypoventilation than the consolidation seen in Panel (a). Dose distribution maps were superimposed on 4DCT and lung ventilation images to visually identify changes on the images and the percentage of the isodose line to which the hypoventilation site corresponded. (c) Axial image of the area where the primary tumor is located in the right upper lobe. The changes on the CT image correspond to the red-dotted 95% isodose line (62.7 Gy). (d) 4DCT ventilation image of the same slice as in Panel (c). The decrease in ventilation is noticeable at the red solid 95% isodose line, but it can also be visualized that it has spread to the yellow solid 30% isodose line (19.8 Gy).

In this case, the Jacobian determinant was used to generate lung ventilation images, where voxels with a large change in volume were considered to have good respiratory function; a value (Vjac) greater than one indicates dilation, and a value less than one indicates contraction. The median Vjac for the whole lung was 1.13 pre-RT, 1.19 at six months post-RT, and 1.14 at one year post-RT. Analysis of the lungs by lobe showed no consistent decline in the right upper lobe, where the primary tumor was located, with Vjac values of at 1.11, 1.16, and 1.13. However, in the left lower lobe, which showed compensatory hypertrophy, there was a tendency for decreases, with Vjac values of 1.24, 1.13, and 1.13, suggesting that hypertrophy may not be directly related to ventilatory function. Changes in lung ventilation in the irradiated field were measured by overlaying the lung ventilation images on the RT plan (Figure 4).

At six months post-RT, CT images showed changes associated with radiation-induced lung injury (RILI) within the 10% isodose line (6.6 Gy), consistent with reduced ventilation. At one year post-RT, CT images showed changes associated with RILI within the 40% isodose line (26.4 Gy), whereas reduced ventilation was observed in the 30% isodose line (19.8 Gy) (Figure 4). The median Vjac for V20 six months post-RT was 0.98, while the median Vjac excluding V20 from the whole lung was 1.19; at one year post-RT, the corresponding values were 0.99 and 1.14.

## Discussion

When considering changes in the respiratory function post-RT for lung cancer, it is important to take into account the pathogenesis of RILI, as it combines acute-phase RP, which occurs within six months of RT, with chronic-phase radiation fibrosis. The extent of RILI is influenced by factors such as lung dose, age, and comorbidities, making it difficult to predict [5]. In this single-case, hypothesis-generating report, we performed quantitative 4DCT analysis to investigate regional changes associated with RILI that may not be fully captured by PFTs. For this patient, the Jacobian determinant from 4DCT was used to generate the lung ventilation images. Previous studies have reported significant correlations between ventilation images generated using 4DCT and V/P SPECT images [4,6]. However, Jacobian-based ventilation estimates are DIR-dependent and may be influenced by methodological choices (e.g., registration settings, phase selection, and breathing irregularity); therefore, the present findings should be interpreted as exploratory.

Analysis of lung dynamics showed different changes inside and outside the irradiated field. A decrease in volume and an increase in CI over time were observed in the right upper and middle lobes, where high lung doses were administered. This may reflect compensatory changes in the non-fibrotic areas of the right upper lobe. In the lung volumes outside the radiation field, the left upper lobe of the lung showed little change, while the left lower lobe of the lung showed an 11% increase one year post-RT compared with the pre-RT volumes. Conversely, there was a minor decrease in the rate of contraction of the enlarged left lower lobe, from 19% to 17%, between pre-RT and one year post-RT. Vjac also slightly decreased from 1.24 to 1.13 in the left lower lobe one year post-RT compared to pre-RT, showing similar results. These results support those of previous studies showing that compensatory hypertrophy post-partial pneumonectomy in adults is due to alveolar expansion rather than lung growth [7]. While compensatory changes after RT have been less frequently quantified at the lobar level in humans, the present observation raises the hypothesis that expansion may preferentially occur toward the diaphragmatic direction, as RT does not create a postoperative cavity space. In the right lower lobe, which received minimal dose except for S6, the CI remained unchanged at 27% across all three time points. It is possible that numerical changes were attenuated by competing mechanisms, including compensatory motion in non-fibrotic areas and contralateral/lobar volume shifts.

A key confounder in this case is the potential overlap between RP and ICI-related pneumonitis. The patient received durvalumab after chemoradiotherapy and developed CTCAE grade 2 pneumonitis treated with steroids. Because RP and ICI pneumonitis can overlap clinically and radiographically, mixed etiologies cannot be excluded; therefore, dose-function interpretations should be made cautiously.

Lung ventilation imaging using the Jacobian determinant function was used to quantitatively investigate the relationship between the lung dose and ventilatory capacity. 4DCT ventilation imaging showed that consolidation associated with RILI coincided with reduced pulmonary ventilation, six months post-RT, whereas one year post-RT, there was reduced ventilation over a wider range than consolidation. Functional imaging modalities (e.g., V/P SPECT) may reveal regional impairment that is not fully captured by anatomic CT changes. In this case, Jacobian-based 4DCT ventilation suggested ventilation reduction extending beyond visible consolidation at one year. Studies using SPECT perfusion imaging have reported a dose-dependent decrease in pulmonary blood flow at low doses of 10-20 Gy [3,8]. To support the dose-function interpretation with minimal quantification, we report plan-based dose metrics (V20 25.1%, V5 46.5%, and mean lung dose 14.42 Gy) and summarize ventilation metrics stratified by dose. Specifically, the median Vjac within the V20 region was 0.98 at six months and 0.99 at one year, whereas the median Vjac in the lung excluding V20 was 1.19 and 1.14, respectively, indicating persistently reduced ventilation in higher dose regions. The decrease in ventilatory capacity with increasing lung dose was similar to that observed in the present case using 4DCT ventilation imaging. In recent years, there have been many reports on the importance of V5 and the absolute volume spared from 5 Gy, in addition to V20, as dosimetric parameters of the lung [5]. In the present case, the Vjac values of irradiated sites > 5 Gy were all < 1, even one year post-RT, indicating the importance of reducing the lung dose in treatment planning. Prospective clinical studies have begun evaluating 4DCT ventilation-guided functional lung avoidance radiotherapy in NSCLC, including a single-arm pilot trial and a phase 2 randomized trial [9,10]. These planning-focused studies complement the present case report, which illustrates longitudinal, dose-overlaid 4DCT ventilation changes during post-RT follow-up in an individual patient.

A limitation of this case report is that PFTs were not performed at the time of post-RT 4DCT imaging; therefore, direct global functional validation of the imaging-derived ventilation metrics was not available. It has been reported that lung doses of 10-20 Gy correlate with FEV1.0, and in the future, PFTs will be performed at the same time as 4DCT imaging [3]. Future studies should incorporate contemporaneous PFTs and/or external functional validation, together with standardized acquisition and DIR settings, to better characterize longitudinal dose-function relationships.

Overall, this single-case analysis suggests that contralateral/lobar volume expansion after RT may not necessarily be accompanied by improved ventilation, and that reduced ventilation can persist within irradiated regions. These findings should be interpreted cautiously given the single-patient design, DIR dependence, potential RP-ICI pneumonitis overlap, and lack of post-treatment PFTs.

## Conclusions

This case illustrates that serial 4DCT-derived ventilation imaging can provide a practical longitudinal view of regional lung function after radiotherapy. Although a non-irradiated lobe showed volumetric enlargement suggestive of compensatory hypertrophy, ventilation metrics did not improve in parallel, indicating a possible dissociation between structural and functional compensation. In contrast, ventilation within the irradiated field showed a persistent dose-associated decline. These findings are hypothesis-generating and may support the integration of functional information with anatomic CT when assessing post-radiotherapy changes, while acknowledging that this is a single case and the results depend on deformable registration. The absence of longitudinal post-treatment PFTs and potential overlap between RP and ICI-related pneumonitis further limit causal inference; therefore, validation in larger cohorts is warranted.

## References

[1] Brunelli A, Kim AW, Berger KI, Addrizzo-Harris DJ (2013). Physiologic evaluation of the patient with lung cancer being considered for resectional surgery: diagnosis and management of lung cancer, 3rd ed: American College of Chest Physicians evidence-based clinical practice guidelines. Chest.

[2] Daly ME, Singh N, Ismaila N (2022). Management of stage III non-small-cell lung cancer: ASCO Guideline. J Clin Oncol.

[3] Weller A, Dunlop A, Oxer A (2019). Spect perfusion imaging versus CT for predicting radiation injury to normal lung in lung cancer patients. Br J Radiol.

[4] Yamamoto T, Kabus S, Lorenz C (2014). Pulmonary ventilation imaging based on 4-dimensional computed tomography: comparison with pulmonary function tests and SPECT ventilation images. Int J Radiat Oncol Biol Phys.

[5] Tsujino K, Hashimoto T, Shimada T (2014). Combined analysis of V20, VS5, pulmonary fibrosis score on baseline computed tomography, and patient age improves prediction of severe radiation pneumonitis after concurrent chemoradiotherapy for locally advanced non-small-cell lung cancer. J Thorac Oncol.

[6] Castillo R, Castillo E, McCurdy M (2012). Spatial correspondence of 4D CT ventilation and SPECT pulmonary perfusion defects in patients with malignant airway stenosis. Phys Med Biol.

[7] Tronc F, Grégoire J, Leblanc P, Deslauriers J (1999). Physiologic consequences of pneumonectomy. Consequences on the pulmonary function. Chest Surg Clin N Am.

[8] Robbins ME, Brunso-Bechtold JK, Peiffer AM, Tsien CI, Bailey JE, Marks LB (2012). Imaging radiation-induced normal tissue injury. Radiat Res.

[9] Baschnagel AM, Flakus MJ, Wallat EM (2024). A phase 2 randomized clinical trial evaluating 4-dimensional computed tomography ventilation-based functional lung avoidance radiation therapy for non-small cell lung cancer. Int J Radiat Oncol Biol Phys.

[10] Yamamoto T, Kabus S, Bal M (2023). Four-dimensional computed tomography ventilation image-guided lung functional avoidance radiation therapy: a single-arm prospective pilot clinical trial. Int J Radiat Oncol Biol Phys.

